# Supplementary material for: Dynamic decision policy reconfiguration under outcome uncertainty
Source: eLife. 2021 Dec 24;10:e65540. doi: 10.7554/eLife.65540 (PMC8806193; doi:10.7554/eLife.65540)
Supplement: Transparent reporting form [file elife-65540-transrepform1.pdf]

eLIFE

1st Floor
24 Hills Road
Cambridge CB2 1JP, UK

P 01223 855340
W elifesciences.org
T @elife

# *eLife's* transparent reporting form

We encourage authors to provide detailed information *within their submission* to facilitate the interpretation and replication of experiments. Authors can upload supporting documentation to indicate the use of appropriate reporting guidelines for health-related research (see EQUATOR Network), life science research (see the BioSharing Information Resource), or the ARRIVE guidelines for reporting work involving animal research. Where applicable, authors should refer to any relevant reporting standards documents in this form.

If you have any questions, please consult our Journal Policies and/or contact us: editorial@elifesciences.org.

**Sample-size estimation**

- You should state whether an appropriate sample size was computed when the study was being designed
- You should state the statistical method of sample size computation and any required assumptions
- If no explicit power analysis was used, you should describe how you decided what sample (replicate) size (number) to use

Please outline where this information can be found within the submission (e.g., sections or figure legends), or explain why this information doesn't apply to your submission:

In Experiment 1, participants completed 600 trials for each of four conditions for a total of 2400 observations per subject. This number of trials was chosen as the result of a power analysis using data simulated from our hypothesized model. Fitting this simulated data with 400-600 trials per condition yielded stable drift-diffusion modeling parameter estimates and recovered the model from which the data were simulated, as measured by higher DIC scores for alternative models (see General statistical analysis section of Methods & Materials section of article; https://github.com/kmbond/loki_0/blob/master/hypotheses.ipynb; and our preregistration, https://osf.io/5esn4). We chose to collect data from 24 participants to fully counterbalance the order of the four conditions.

In Experiment 2, four participants completed 400 trials for each of nine conditions for a total of 3600 observations per subject. This number of trials per condition was based on the original power analysis described above while giving consideration to the amount of time participants were required to commit to the experiment and the extended intertrial intervals necessary to collect clean estimates of the pupillary response. Here, we chose to collect data from four participants to test the replicability of the effects observed in Experiment 1 (see Methods and Materials section of article for more detail).

**Replicates**

- You should report how often each experiment was performed
- You should include a definition of biological versus technical replication
- The data obtained should be provided and sufficient information should be provided to indicate the number of independent biological and/or technical replicates
- If you encountered any outliers, you should describe how these were handled
- Criteria for exclusion/inclusion of data should be clearly stated
- High-throughput sequence data should be uploaded before submission, with a private link for reviewers provided (these are available from both GEO and ArrayExpress)


eLife Sciences Publications, Ltd is a limited liability non-profit non-stock corporation incorporated in the State of Delaware, USA, with company number 5030732, and is registered in the UK with company number FC030576 and branch number BR015634 at the address 1st Floor, 24 Hills Road, Cambridge CB2 1JP | August 2014

Please outline where this information can be found within the submission (e.g., sections or figure legends), or explain why this information doesn't apply to your submission:

Replication involved exposing different participants to the same experimental conditions (e.g. biological replication). Information regarding replication can be found in the Stimuli and Procedures subsection of Methods and Materials. Outlier exclusion methods are described in the same section.

**Statistical reporting**

- Statistical analysis methods should be described and justified
- Raw data should be presented in figures whenever informative to do so (typically when N per group is less than 10)
- For each experiment, you should identify the statistical tests used, exact values of N, definitions of center, methods of multiple test correction, and dispersion and precision measures (e.g., mean, median, SD, SEM, confidence intervals; and, for the major substantive results, a measure of effect size (e.g., Pearson's r, Cohen's d)
- Report exact p-values wherever possible alongside the summary statistics and 95% confidence intervals. These should be reported for all key questions and not only when the p-value is less than 0.05.

Please outline where this information can be found within the submission (e.g., sections or figure legends), or explain why this information doesn't apply to your submission:

Statistical methods are described and justified in the Analyses subsection of Methods and Materials in the article. Statistical estimates, their 95% confidence intervals, and exact p-values for all analyses are reported in the Results section.

(For large datasets, or papers with a very large number of statistical tests, you may upload a single table file with tests, Ns, etc., with reference to sections in the manuscript.)

**Group allocation**

- Indicate how samples were allocated into experimental groups (in the case of clinical studies, please specify allocation to treatment method); if randomization was used, please also state if restricted randomization was applied
- Indicate if masking was used during group allocation, data collection and/or data analysis

Please outline where this information can be found within the submission (e.g., sections or figure legends), or explain why this information doesn't apply to your submission:

For both experiments, the sequence of experimental conditions was pseudorandomized and assigned to each participant number. The experimenter was not blind to group allocation or the experimental condition during data collection and data analysis. Participants were naive to the experimental condition during data collection.

**Additional data files ("source data")**


eLife Sciences Publications, Ltd is a limited liability non-profit non-stock corporation incorporated in the State of Delaware, USA, with company number 5030732, and is registered in the UK with company number FC030576 and branch number BR015634 at the address 1st Floor, 24 Hills Road, Cambridge CB2 1JP | August 2014

- We encourage you to upload relevant additional data files, such as numerical data that are represented as a graph in a figure, or as a summary table
- Where provided, these should be in the most useful format, and they can be uploaded as "Source data" files linked to a main figure or table
- Include model definition files including the full list of parameters used
- Include code used for data analysis (e.g., R, MatLab)
- Avoid stating that data files are "available upon request"

Please indicate the figures or tables for which source data files have been provided:

Behavioral data and their computational derivatives are available at https://github.com/kmbond/dynamic_decision_policy_reconfiguration. Code used to generate all figures can be found here: https://github.com/kmbond/dynamic_decision_policy_reconfiguration/tree/master/figure_nbs. Model estimation scripts can be found here: https://github.com/kmbond/dynamic_decision_policy_reconfiguration/tree/master/analysis. Raw pupillometry data (DOI: 10.1184/R1/13543133), the features of the task-evoked pupillometry response (DOI: 10.1184/R1/13543067), and the principal components calculated from those features (DOI: 10.1184/R1/13543160) are available at https://kilthub.cmu.edu/projects/Dynamic_decision_policy_reconfiguration_under_outcome_uncertainty/96116.


eLife Sciences Publications, Ltd is a limited liability non-profit non-stock corporation incorporated in the State of Delaware, USA, with company number 5030732, and is registered in the UK with company number FC030576 and branch number BR015634 at the address 1st Floor, 24 Hills Road, Cambridge CB2 1JP | August 2014
